# Liposome-based RNAi delivery in honeybee for inhibiting parasite *Nosema ceranae*

**DOI:** 10.1016/j.synbio.2024.07.003

**Published:** 2024-07-18

**Authors:** Yue Qi, Chen Wang, Haoyu Lang, Yueyi Wang, Xiaofei Wang, Hao Zheng, Yuan Lu

**Affiliations:** a College of Food Science and Nutritional Engineering, China Agricultural University, Beijing, 100083, China; b Department of Chemical Engineering, Tsinghua University, Beijing, 100084, China; c Key Laboratory of Industrial Biocatalysis, Ministry of Education, Tsinghua University, Beijing, 100084, China

**Keywords:** Honeybee, Liposome, *Nosema ceranae*, RNA interference

## Abstract

*Nosema ceranae*, a parasite that parasitizes and reproduces in the gut of honeybees, has become a serious threat to the global apiculture industry. RNA interference (RNAi) technology can be used to inhibit *N. ceranae* growth by targeting silencing the thioredoxin reductase (TrxR) in *N. ceranae*. However, suitable carriers are one of the reasons limiting the application of RNAi due to the easy degradation of dsRNA in honeybees. As a vesicle composed of a lipid bilayer, liposomes are a good carrier for nucleic acid delivery, but studies in honeybees are lacking. In this study, liposomes were used for double-stranded RNA (dsRNA) dsTrxR delivery triggering RNAi to inhibit the *N. ceranae* growth in honeybees. Compared to naked dsTrxR, liposome-dsTrxR reduced *N. ceranae* numbers in the midgut and partially restored midgut morphology without affecting bee survival and gut microbial composition. The results of this study confirmed that liposomes could effectively protect dsRNA from entering the honeybee gut and provide a reference for using RNAi technology to suppress honeybee pests and diseases.

## 1 Introduction

Honeybees (*Apis mellifera*) are widely recognized as one of the important pollinators on earth that can promote plant reproduction by spreading pollen between flowers, which is important for maintaining ecological balance and advancing agricultural production. In recent years, however, honeybees have been greatly threatened by invasive parasites and pathogens, habitat loss, pesticide use, and climate changes [1,2]. Honeybees are susceptible to various pathogens and pests, including bacteria, fungi, mites, viruses, and protozoans [3,4].

Microsporidia are obligate intracellular fungal parasites that can form spores and parasitize the epithelial cells of the midgut of honeybees, absorbing and utilizing the nutrients in the honeybee's gut to grow and reproduce, and disrupting the honeybee's ability to ingest energy normally [3,5]. Microsporidian infection is an important disease that jeopardizes the beekeeping industry, leading to reduced honeybee vigor, decreased colony honey production, and may even lead to the collapse of the entire colony with Colony Collapse Disorder (CCD) [6,7]. Common microsporidian infections in honeybees include isolated and mixed infections of *Nosema ceranae* and/or *Nosema apis*. The current study found that *N. ceranae* is more widespread in honeybee colonies around the world [[8], [9], [10], [11]].

Control methods for *N. ceranae* in honeybees include fumagillin, natural extracts such as propolis, small molecule actives, and RNA interference [12]. Among them, RNA interference (RNAi) is a sequence-specific small-molecule non-coding RNA-induced gene silencing [13], and RNAi technology has been widely used in the field of crop pest control due to its high specificity and low environmental pollution. It has been demonstrated that RNAi-induced reductions in gene expression are important for inhibiting *N. ceranae* growth or modulating honeybee immunity, with the potential to control *N. ceranae* infections [[14], [15], [16]]. Honeybees can remove invasive parasites by triggering an oxidative stress response. Previous studies have demonstrated that *N. ceranae* can alleviate bee-induced oxidative stress through the glutathione system, and therefore the knockout of the thioredoxin reductase gene could be used to inhibit *N. ceranae* growth [14]. The use of RNAi to inhibit *N. ceranae* mainly depends on the RNAi delivery method, the stability of RNAi, and target genes, so the efficient delivery of exogenous double-stranded RNAs (dsRNA) triggering RNAi has become an important challenge in the control of *N. ceranae*.

Currently, commonly used insect dsRNA delivery includes microinjection, transgenic plants, and soaking [17]. Nanoparticle-mediated dsRNA delivery is a promising dsRNA delivery method. Liposomes are defined as phospholipid vesicles consisting of one or more lipid bilayers with the unique ability to encapsulate both hydrophobic and hydrophilic drugs [18]. As a drug delivery system, liposomes offer a variety of advantages, including biocompatibility, the ability to carry hydrophilic and hydrophobic drugs, and biodegradability [19]. Liposomes can form lipid complexes with dsRNA by electrostatic interaction. Liposome delivery of dsRNA has been partially used in insects, *e.g.*, *Spodoptera frugiperda* [20], fruit flies [21], cockroaches [22], but more research is still needed to determine its applicability and effectiveness in different insect species.

**Fig. 1:**
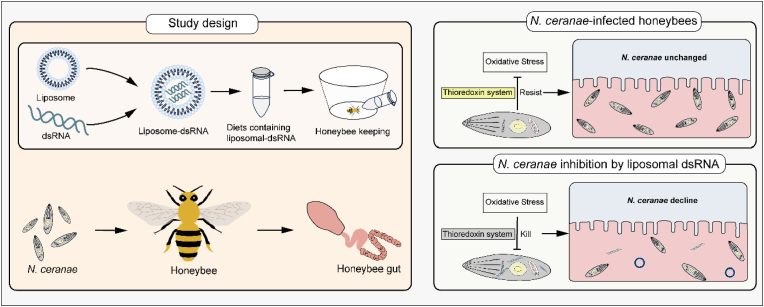
Schematic research diagram of liposome-based RNAi delivery in honeybee for inhibiting parasite *N*. *ceranae*.

In this study, liposomes were used to protect dsTrxR to inhibit *N. ceranae* in honeybees, and using liposomes as a delivery material in insect RNAi was explored (Fig. 1). Liposomes could load dsRNA, avoiding the effects of honeybee midgut pH and nuclease, and reach the honeybee gut to interact with *N. ceranae* without difficulty. Artificial feeding infected honeybees with *N. ceranae*, and then measured the number of *N. ceranae* in the midgut of honeybees and the expression of *N. ceranae* genes, confirming the protective effect of liposomes on dsRNA. The gut was the site of the direct presence of liposomes and *N. ceranae*, verifying that this process did not significantly affect the gut microbiota. This study could provide a new technique for controlling *N. ceranae* in honeybees and build a basis for applying RNAi technology in insects.

## 2 Materials and methods

### 2.1 Materials

Primers (Table S1, Supporting Information) were synthesized by Sangon Biotech (China). Percoll solution used for isolation and purification of *N. ceranae* was purchased from Solarbio (China). FastPure EndoFree Plasmid Mini Kit for plasmid extraction, pCE2-TA-Blunt-Zero, FastT1 chemoreceptor cells for the construction of plasmids for *in vitro* dsRNA synthesis, RNA easy Isolation Reagent for RNA extraction, HiScript III All-in-one RT SuperMix Perfect for qPCR, and ChamQ Universal SYBR qPCR Master Mix for qPCR were purchased from Vazyme Biotch (China). MEGAscript RNAi Kit for dsRNA *in vitro* transcription was purchased from Ambion (USA). Liposomes and Opti-MEM™ were purchased from Thermo (USA). The Gene ID of TrxR was 36320965.

### 2.2 Honeybee feeding

The honeybees (*A. mellifera*) used in this experiment were obtained from the experimental apiary of China Agricultural University. The honeybees used in the experiments were collected on day 1 post-feathering and kept in an incubator (Bluepard, China) at a temperature of 36 °C and a humidity of 50 %. During the experimental period, the honeybees were fed a diet of pollen granules with 50 % sucrose solution (wt/vol), and the diet was ensured to be adequate. To collect the honeybee samples, sterilized forceps were used to remove the honeybee guts. There are no ethical rules for experiments on bees.

### 2.3 *N. ceranae* spore purification

*N. ceranae* spores were collected from heavily infected worker honeybees. The worker honeybees were placed on ice to render them unconscious, then the midgut of the honeybee was removed using sterilized forceps. The midgut was placed in distilled water and ground thoroughly using a grinder. The filtrate was centrifuged at 3000 g for 5 min at 4 °C, the supernatant was discarded, and the precipitate was resuspended in distilled water. The spore suspension was then covered with a gradient Percoll solution over a gradient of 25 %, 50 %, 75 %, and 100 %, and centrifuged at 5000 g for 15 min at 4 °C. The supernatant was discarded, and the white precipitate was resuspended in sterile distilled water and stored at 4 °C (Fig. S1, Supporting Information) [14]. The *N. ceranae* spores used for each infection were isolated and purified the day before. The identity of the isolated *N. ceranae* was determined by amplification with specific primers.

### 2.4 *N. ceranae* spore infection

To control for the same number of *N. ceranae* spores infecting each honeybee, each honeybee was fed the same amount of *N. ceranae* spores. Each honeybee was starved for 2 h and then given 2 μl of sucrose solution (50 % wt/vol) containing 10^5^ *N. ceranae* spores (Fig. S2, Supporting Information). The number of spores in the midgut of infected honeybees was counted after 14 days. The honeybee midgut was removed with sterilized forceps and placed in 100 μl of sterile distilled water and ground well. 5 μl of the midgut suspension was taken on a hemocytometer and counted using an optical microscope at 400 × .

### 2.5 dsRNA synthesis and delivery

To synthesize dsRNA *in vitro*, the gene coding region of the *N. ceranae* cDNA was amplified using forward and reverse primers containing the T7 promoter. The amplified gene fragments were cloned into the pCE2-TA-Blunt-Zero vector and verified by Sanger sequencing. The TrxR fragment was amplified by PCR using the obtained plasmid as a template with primers with T7 promoter. The amplification results were confirmed by agarose gel electrophoresis. The dsRNA was synthesized *in vitro* using the MEGAscript RNAi Kit, and 1 μg of PCR product was used as the transcription template. To ensure the synthesis of dsRNA, enzyme-free reagents and equipment were used and operated on an ultra-clean bench, and the experimental staff wore masks and gloves throughout. The obtained dsRNA was resuspended in RNAase-free water. The concentration was detected by NanoDrop 8000 spectrophotometer (Thermo Fisher Scientific), and the integrity was detected by agarose gel electrophoresis. The fractionated dsRNA was stored at −80 °C before use.

Liposomes were used as carriers to protect dsRNA from degradation. Liposomes were obtained by mixing 2 μl of liposome with 25 μl of Opti-MEM™ for 10 min according to the manufacturer's protocol, while quantitative dsRNA was diluted in the same volume of Opti-MEM™ solution, and subsequently, the two solutions were mixed for 5 min to obtain the liposome-dsRNA mixture, which was ready to be used every day (Fig. S3, Supporting Information). dsRNA was prepared at a final concentration of 5 ng/μl, and each honeybee ingested about 200 ng of dsRNA per day.

### 2.6 Characterization of liposome

Liposomes were analyzed for particle size and potential using Malvern particle sizer. The morphology of liposomes was observed using transmission electron microscopy (JEM2010).

### 2.7 RNA isolation and qPCR for quantification of *N. ceranae*

Honeybee midguts were snap-frozen in liquid nitrogen immediately after collection and stored at −80 °C before extraction. Midgut RNA from individual honeybees was extracted using RNA easy Isolation Reagent, and the purity and quantity of RNA samples were determined using a NanoDrop 8000 spectrophotometer (Thermo Fisher Scientific). Subsequently, cDNA was synthesized using HiScript III All-in-one RT SuperMix Perfect for qPCR.

Quantitative real-time PCR was performed in standard 96-well plates using ChamQ Universal SYBR qPCR Master Mix and QuantStudio 1 Real-Time PCR Instrument (Thermo Fisher Scientific, Waltham, MA, USA). The reaction system was 10 μl Mix, 0.4 μl forward primer (10 μM), 0.4 μl reverse primer (10 μM), 0.2 μl cDNA and 9 μl DEPC water. The reaction program was incubation at 95 °C for 3 min, denaturation at 95 °C for 40 cycles of 10 s, and annealing/extension at 60 °C for 20 s. *N. ceranae* TrxR and actin primers were obtained from Lang et al. [14]. The relative expression of TrxR was calculated by the 2^-ΔΔCT^ method using the actin gene of *N. ceranae* as a control [23].

### 2.8 Hematoxylin and eosin staining (H&E staining) of honeybee midgut

The gut of the honeybees was removed using sterilized forceps, and only the midgut was retained. The samples were fixed using 4 % paraformaldehyde for 24 h, then dehydrated with an ethanol gradient, and then embedded in paraffin wax. Subsequently, the paraffin-embedded samples were transversely cut and stained with hematoxylin and eosin. Images of the midgut samples were obtained using an optical microscope.

### 2.9 Honeybee gut microbial analysis

The gut of honeybees on day 14 was collected and submitted to Novogene Company (Beijing, China) for DNA extraction and 16S ribosomal RNA (rRNA) sequencing of the V3–V4 region. After obtaining the sequencing results, bioinformatics analysis was performed using QIIME2 [24]. The raw data were merged with bipartite sequences and denoised using Dada2 [25], and the honeybee gut microbial database was used to annotate the obtained data. Gut classification information and abundance information were obtained and then visualized in R using file2meco [26].

### 2.10 Statistical analysis

Statistical analyses were performed and plotted using GraphPad Prism 5.0 (GraphPad, San Diego, CA, USA). Kruskal-Wallis test in one-way ANOVA was used to detect the number of *N. ceranae* and gene expression levels among different groups.

## 3 Results

### 3.1 Characterization of liposome morphology

**Fig. 2:**
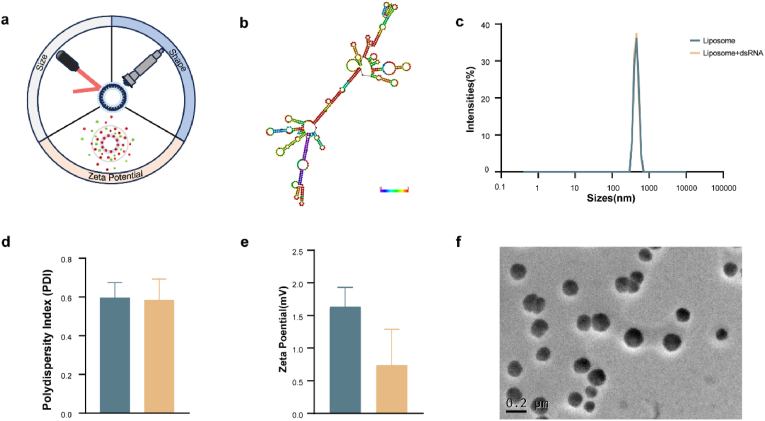
Liposome characterization. (a) Schematic representation of liposome characterization. (b) Secondary structure of dsRNA predicted by RNA fold (http://rna.tbi.univie.ac.at/cgi-bin/RNAWebSuite/RNAfold.cgi). (c) Particle size of liposomes. (d) Polydispersity index (PDI) of liposomes. Liposome: dark cyan; Liposome-dsRNA: light brown. (e) Zeta potential of liposomes. Liposome: dark cyan; Liposome-dsRNA: light brown. (f) TEM image of liposomes.

Characterization of liposomes is an important part of assessing the physicochemical properties of liposomes. Among them, particle size, zeta potential and shape, as important metrics in the characterization of liposomes, correlate with liposome stability and drug delivery (Fig. 2).

To understand the potential of liposomes as dsRNA delivery vehicles, the particle size, zeta potential, and shape were determined. The particle sizes of liposomes and liposome-dsRNA mixture were measured using dynamic light scattering (DLS), and the results showed a single peak distribution. The particle size distribution ranged from 300 nm to 600 nm, with an average particle size of 468 ± 108 nm. The zeta potential is another important property to characterize liposomes. The potential of liposomes was 1.634 mV, and the potential of liposome-dsRNA was 0.739 mV. The morphology and structure of liposomes were observed by transmission electron microscopy, and it was found that the liposomes were distributed in a spherical shape. The size of the liposomes was observed to be about 180 nm in transmission electron microscopy, which was smaller than the DLS results, which may be due to different measurement mechanisms.

### 3.2 Inhibiting *N. ceranae* by liposome delivery of dsTrxR in honeybees

**Fig. 3:**
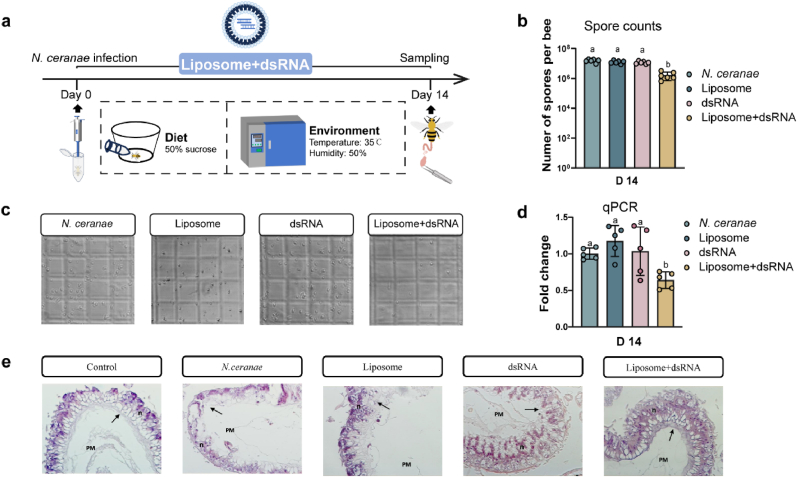
*In vivo* experiments in honeybees. (a) Experimental design of liposomal delivery of dsRNA to inhibit *N. ceranae* in honeybees. (b) Number of *N. ceranae* in different groups, the letter above each bar represents the statistical difference between the treatments (p < 0.05). (c) Quantification of *N. ceranae* number by hemocytometer under a 400 × light microscope. (d) Relative expression of *N. ceranae* TrxR gene, the letter above each bar represents the statistical difference between the treatments (p < 0.05). (e) Representative pictures of honeybee midgut stained by H&E. Arrow symbol, epithelial cell. n, nucleus. PM, peritrophic matrix.

RNAi efficiency needs to be further evaluated in honeybees. On day 1, honeybees were infected with *N. ceranae*; then, the mixture of liposomes and dsTrxR was added to the honeybee diet over the following 14 days, and the liposome-dsRNA mixture was introduced into the honeybee gut by oral delivery. On day 14, the midgut of the honeybees was collected (Fig. 3a).

To verify the inhibitory effect of liposome-delivered dsTxR on *N. ceranae*, experiments were performed in honeybees. According to the honeybee experiments, liposomes did not significantly affect bee survival (Fig. S4, Supporting Information). *N. ceranae* counts are a direct indicator of delivery efficacy and are counted through a microscope. The counting results showed that the liposome-dsTrxR mixture could effectively reduce the number of *N. ceranae* in the honeybee gut from 10^7^ to 10^6^ compared to the liposome and dsRNA groups (Fig. 3b and c). Expression of the TrxR in *N. ceranae* was calculated by RT-qPCR. RT-qPCR results showed that TrxR gene expression of *N. ceranae* was decreased in the liposome-dsTrxR group, confirming that the decrease in the number of *N. ceranae* was due to the reduced expression of TrxR of *N. ceranae* (Fig. 3d). The midgut is the main site of *N. ceranae* aggregation, and *N. ceranae* affects the morphology of the honeybee midgut. It was observed by H&E staining that *N. ceranae* resulted in fragmentation of cell membranes, displacement of nuclei and disruption of peritrophic matrix arrangement in the honeybee midgut. When the number of *N. ceranae* reduced, the morphology of the midgut was restored, but the peritrophic matrix arrangement was still disrupted (Fig. 3e). Liposomes could successfully deliver dsTrxR to the honeybee midgut by oral delivery to inhibit the growth of *N. ceranae*. This proved liposomes could successfully deliver dsTrxR to the honeybee gut by oral delivery to inhibit the growth of *N. ceranae*.

### 3.3 Effects of liposome delivery of dsTrxR on the gut microbiota of the honeybee

**Fig. 4:**
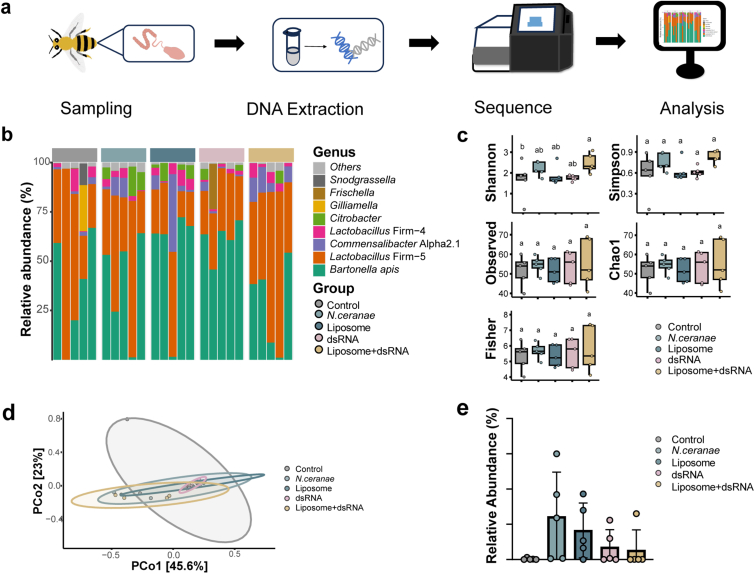
Honeybee gut microbiota composition and diversity. (a) Genus-level relative abundance. (b) α diversity. (c) β diversity. (d) *Citrobacter* content.

Gut microbes have a significant impact on the physiological health and immune system of honeybees. Gut microbes can be affected by various factors, including disease and nanomaterials. Some studies have shown that honeybee gut microbiota can provide some protection against *N. ceranae.* At the same time, liposomes may affect gut microbes. 16S sequencing of 14-day honeybee gut samples was performed to investigate the effect of liposomes on *N. ceranae*-infected honeybee gut microbiota (Fig. 4a).

The effects of liposomes and *N. ceranae* on gut microbiota were determined to analyze the feasibility of liposomes as RNAi delivery materials for honeybees. At the genus level, the dominant bacteria in the gut microbes of 14-day-old honeybees were *Bartonella apis* and *Lactobacillus* Firm-5 (Fig. 4b). The gut microbiota composition varied somewhat among individual honeybees. α diversity reflects the species richness and diversity of microbiota. As shown in Fig. 4c, except for the Shannon index, there were no significant differences in α diversity among the different groups. β diversity referred to the variability in the microbial composition among samples, and there was no significant separation among different subgroups, as shown in Fig. 4d, which showed that *N. ceranae* and liposome did not have a significant effect on the gut microbiota of honeybees. *Citrobacter* is an environmental bacterium in the gut of honeybees, and although there was no significance, compared to the very low *Citrobacter* abundance in controls, *N. ceranae* infection increased *Citrobacter* abundance in the gut, whereas liposome-dsRNA mixture decreased *Citrobacter* abundance (Fig. 4e). Compared to control, *N. ceranae* and liposome produced less effect on the gut microbiota of honeybees, and *Citrobacter* could be used as a microbial indicator of *N. ceranae* infection.

## 4 Discussion

### 4.1 Morphological characterization of liposomes

Liposomes have been widely used as excellent delivery carriers for a variety of drugs. Characterization of liposomes is useful to understand the properties of liposomes to ensure their applications. Liposomes were characterized for size, zeta potential, shape, laminar phase behavior, encapsulation, efficiency, and drug release [27]. In this study, the size, zeta potential, and shape of liposomes were determined.

Liposome size is an important metric in characterizing liposomes and correlates with their distribution and uptake. In this study, the average particle size of liposomes determined by DLS was 468 nm, and that of liposomes determined by TEM was 170 nm. DLS calculates the average particle size by analyzing the change in scattered light caused by the Brownian motion of dispersed particles in solution [28], while TEM calculates particle size by acquiring images through electrons transmitted through the sample [29]. Differences in measurement principles between the two techniques lead to different results. The potential of a liposome is the total net charge on the surface of the liposome and is a fundamental indicator of the control of electrostatic interactions between suspended particles, which is usually correlated with the stability of the liposome in its environment [30,31]. The net charge of liposomes is influenced by various factors, including liposome composition, paper head genes, and the external environment [27]. The shape of liposomes is an important indicator for characterizing liposomes and correlates with their stability. The shape of liposomes is generally determined by microscopy, which allows observation of the morphology of individual particles of liposomes. The stability of liposomes is an important metric for assessing liposome applications. As a vesicle composed of lipid bilayers, liposomes are considered a moderately unstable colloidal system. The current study confirms that liposome-dsRNA mixtures can be stored for long periods of more than 30 days at low temperature, and that partial aggregation of liposomes may occur with increasing temperature and storage time [32]. The stability of liposomes will also be enhanced in the future by optimizing liposome formulations and improving liposome preparation.

### 4.2 Effective improvement of dsRNA delivery by liposomes in honeybees

*N. ceranae* is a widely distributed microsporidian that has become a global threat to honeybees since it was first detected in the Asian honeybee *Apis cerana* in 1994 [33]. In recent years, studies have sequenced the *N. ceranae* genome, making the use of RNAi technology a new approach to suppressing *N. ceranae* [34]. Compared to other methods of *N. ceranae* control, RNAi is a natural anti-infection mechanism of the honeybee immune response [35], which is more effective and less likely to cause antibiotic residues. Several studies have been conducted to knock down the expression of *N. ceranae* genes, such as polar tubulin (PTP) [36], and spore wall proteins (SWP) [16]. Lang et al. found that *N. ceranae* could maintain survival against the host's own oxidative stress through the thioredoxin and glutathione systems, and found that knocking down the γ-glutamylcysteine synthetase and thioredoxin reductase genes of *N. ceranae* could reduce the number of *N. ceranae* in infected honeybees [14].

In this study, liposomes effectively protected dsRNA from entering the honeybee gut, and liposomal dsRNA delivery resulted in a 10-fold reduction of *N. ceranae* from 10^7^ to 10^6^ in the honeybee midgut. No significant effect of *N. ceranae* on the survival of bees was found in this experiment, but it was similar to the results of some studies [[37], [38], [39]]. The colony collapsed due to *N. ceranae*, which was confirmed in the strict absence of mites [40]. Since the bees in the experiment were collected in an apiary, it was difficult to avoid mites. Fumagillin proved effective in treating *N. ceranae* infections in several studies [41]. In cage experiments, the use of fumagillin reduced the number of *N. ceranae* in the range of 2 × 10^6^ to 1 × 10^6^ [42]. In field experiments, fumagillin temporarily reduced the number of *N. ceranae*, but there was no significant difference between the treatment and control groups over time [43]. Although fumagillin can inhibit the growth of *N. ceranae*, they can cause some damage to bees, such as altering the ultrastructure of hypopharyngeal glands and metabolic proteins [44]. *N. ceranae* have shown some degree of resistance to fumagillin, and it has been found that fumagillin may even lead to overproliferation of *N. ceranae* [45,46]. Fumagillin are also toxic to humans and may cause chromosomal aberrations and induce cancer [47]. Residues of fumagillin can remain in hive products, posing a health risk to humans and bees, and have been banned in many countries, including the EU [47]. A variety of natural products have also been attempted to inhibit *N. ceranae*, such as essential oils, plant extracts, thymol, and propolis. The natural extracts showed an inhibitory effect on *N. ceranae*. Andrographis paniculata extract reduced *N. ceranae* from 4 × 10^8^ to 1.48 × 10^8^ [48]. Thymol reduced *N. ceranae* from 1.3 × 10^5^ to 2.8 × 10^4^. There are also some natural products that inhibit *N. ceranae* but cause an increase in bee mortality, such as essential oils extracted from propolis and radish sulfide [37,49]. Natural products as a potential method of *N. ceranae* control, further studies are still needed to assess their effectiveness and their toxicity [50]. RNAi is a more promising method of inhibiting *N. ceranae* than before. The efficiency of RNAi against *N. ceranae* is correlated with the number of initial *N. ceranae* infections, the number of dsRNA, and gene stability [16,36].

As a common reverse genetics tool, RNAi is now widely used in the insect field, especially for pest control. However, a major challenge that RNAi needs to overcome is the stability of dsRNA triggered for RNAi. Despite the high efficiency of injectable delivery, it is time-consuming and complicated due to the small size of the insect [51]. Fed delivery is a gentle and non-invasive method of dsRNA delivery. dsRNA ingested by feeding needs to pass through the gut before being taken up by the cells, where nuclease and pH can affect the stability of dsRNA. Several studies have confirmed that dsRNases in the midgut of insects cause dsRNA degradation [26,52], but as of now, there is still no specialized study on the degradation of dsRNA by dsRNases in the midgut of the honeybee. RNA is most stable in solutions at pH 4.0–5.0 and is susceptible to alkaline degradation when pH > 6 [53]. In contrast, the midgut pH of honeybees with traditional gut communities reaches up to 7.0, so dsRNA is highly susceptible to degradation in the honeybee midgut [54]. This study suggests that liposomes can be used as a material to encapsulate dsRNA and effectively reduce the degradation of dsRNA by nuclease and pH in the honeybee gut. Compared to naked dsTrxR delivery, the liposome and dsTrxR mixture group significantly reduced the number of *N.ceranae* in the honeybees and suppressed *N.ceranae* gene expression. In this study, TrxR gene expression was increased in some individuals in the liposome and dsRNA groups. The increase may be due to *N. ceranae* trying to defend against oxidative stress. Both liposomes and dsRNA are small molecules that can enter *N. ceranae* and perhaps affect the oxidative stress response of *N. ceranae*, leading to increased TrxR gene expression in some individuals. The effects of liposomes and dsRNA on *N. ceranae* still need to be evaluated in subsequent studies.

In recent years, to improve the stability of orally delivered dsRNA, researchers have tested various delivery materials for use in insect nucleic acid delivery, such as chitosan, cationic polymers, and liposomes. Chitosan is the first nanomaterial to be used in insect RNAi, and chitosan-mediated oral delivery of dsRNA can significantly improve RNAi efficiency [55]. However, chitosan binds to negatively charged proteins and, therefore, requires structural modification [56]. Cationic dendritic polymers are good dsRNA carriers due to high water solubility. Yin and Shen utilized cationic core-shell fluorescent nanoparticles to construct star-shaped polycations as a type of cationic dendritic polymer [55], which have been used in RNAi in a variety of insects [[57], [58], [59]], but cytotoxicity limits its application. Liposomes, as a highly biocompatible material, have been used to deliver dsRNA [32]. Liposomes consist of a lipid bilayer made up of phospholipids that can release encapsulated drugs into the cell by fusing with the cell membrane after entering the cell [60,61]. The nucleic acid loading efficiency of liposomes can be improved by the addition of cholesterol, 1,2-dioleoyl-*sn*-glycerol-3-phosphoethanolamine, and others [62]. In addition, liposomes are easy to prepare and have good application prospects. Liposomal delivery of dsRNA has been tentatively explored in other insects, but no studies have explored the use of liposomal delivery of dsRNA in honeybees. This study confirms the effectiveness of liposomes for dsRNA delivery in honeybees. Delivery of dsRNA in liposomes can effectively counteract multiple threats to honeybees, improve bee health, and increase productivity. As *N*. *ceranae* is an intracellular parasite that specializes in infesting bees, they are only able to reproduce in the epithelial cells of the midgut of bees [63,64]. The lack of suitable honeybee midgut epithelial cell lines limits the *in vitro* validation of RNAi technology.

### 4.3 No significant effect on the microbial composition of the honeybee gut by liposomes

The microbiota of honeybees includes *Lactobacillus* Firm-5, *Lactobacillus* Firm-4, *Bifidobacterium*, *Snodgrassella*, *Gilliamella*, *Frischella*, *Bartonella,* and *Commensalibacter* [65]. Gut microbes are intrinsically linked to honeybee health, including transduction of endocrine signals, stimulation of the immune system, and protection against pathogens [66]. Honeybee gut microbiota may be affected by a variety of external factors, including seasonal changes [67], pathogens and parasite infection [68], and the use of pesticides and antibiotics [69,70].

In this study, the main dominant microbiota in the honeybees were *Bartonella* and *Lactobacillus* Firm-5, which may be related to the caging method used in the experiment. Alberoni et al. noted that caged honeybees could be deficient in γ-proteobacteria, including *Frischella*, *Gilliamella*, and *Snodgrassella*. There was also an increase in the abundance of the environmental bacteria *Citrobacter*, *Cosenzaea,* and *Morganella* and the pathogenic bacterium *Serratia* [68]. Several studies have examined the role of honeybee gut microbiota in resistance to *N. ceranae* infections. Supplementation with probiotics and prebiotics can reduce the number of *N. ceranae* [71,72]. An increase in *Citrobacter* in the gut of honeybees infected with *N. ceranae* was also found in this study, although there was no statistical difference. However, *N. ceranae* infections do not cause major compositional changes in gut microbes. This study supports this conclusion. The gut of honeybees consists of crop, midgut, ileum, and rectum, and the distribution of gut microbiota in honeybees varies in different parts of the gut, mainly concentrated in the ileum and rectum [65]. In contrast, *N. ceranae* is mainly present in the midgut, which reduces the interaction between *N. ceranae* and gut microbes. In the future, it may be necessary to focus on the midgut if one wants to explore the interactions between gut microbes and *N. ceranae*.

## 5 Conclusion

This study attempts to apply liposomal delivery of dsRNA in honeybees to explore the potential of liposome-mediated RNAi systems for application in honeybees. Preliminary exploration of the delivery of dsTrxR, a gene that inhibits the growth of *N. ceranae* in the gut of honeybees, using liposomes. It has demonstrated that liposomes as a nanomaterial can effectively prevent the degradation of dsRNA by gut pH and nuclease, so that the dsRNA can reach the target location to inhibit the growth of *N. ceranae*. Liposomes could be taken orally, a non-invasive way to reach the bee gut, reducing their impact on the honeybees. Compared to naked dsRNA, liposomes reduced the number of *N. ceranae* in honeybees by 10-fold. It was also found that neither liposomes nor *N. ceranae* had a significant effect on the gut microbiota, although they were directly present in the honeybee gut.

In the future, attention will be paid to the regulation of liposome structure and the use of light, magnetism, and other technologies to achieve precise targeting and efficient delivery of liposomes. The use of liposomal delivery of dsRNA could be a promising RNAi delivery method for further applications in insect pest suppression for greater environmental and agricultural value.

## Data availability

Data will be made available on request.

## CRediT authorship contribution statement

**Yue Qi:** Conceptualization, Validation, Visualization, Investigation, Data curation, Writing – original draft. **Chen Wang:** Conceptualization, Validation, Visualization, Investigation, Data curation, Writing – original draft. **Haoyu Lang:** Validation, Visualization, Investigation, Data curation. **Yueyi Wang:** Validation, Visualization, Investigation, Data curation. **Xiaofei Wang:** Conceptualization, Funding acquisition, Resources, Supervision, Writing – review & editing. **Hao Zheng:** Conceptualization, Funding acquisition, Resources, Supervision, Writing – review & editing. **Yuan Lu:** Conceptualization, Funding acquisition, Resources, Supervision, Writing – review & editing.

## Declaration of competing interest

The author is an Editorial Board Member/Editor-in-Chief/Associate Editor/Guest Editor for [*this journal* (Synthetic and Systems Biotechnology)] and was not involved in the editorial review or the decision to publish this article.
