# Supplementary material for: Liposome-based RNAi delivery in honeybee for inhibiting parasite Nosema ceranae
Source: Synth Syst Biotechnol. 2024 Jul 18;9(4):853–60. doi: 10.1016/j.synbio.2024.07.003 (PMC11320372; doi:10.1016/j.synbio.2024.07.003)
Supplement: Multimedia component 1 [file mmc1.docx]

**Supporting Information**

**Liposome-based RNAi delivery in honeybee for inhibiting parasite *Nosema ceranae***

Yue Qi, Chen Wang, Haoyu Lang, Yueyi Wang, Xiaofei Wang*, Hao Zheng*, Yuan Lu*

Yue Qi, Haoyu Lang, Yueyi Wang, Xiaofei Wang, Hao Zheng

College of Food Science and Nutritional Engineering, China Agricultural University, Beijing 100083, China

Yue Qi, Chen Wang, Yueyi Wang, Yuan Lu

Department of Chemical Engineering, Tsinghua University, Beijing 100084, China

Key Laboratory of Industrial Biocatalysis, Ministry of Education, Tsinghua University, Beijing 100084, China

* Correspondence:

Xiaofei Wang, xiaofei.wang@cau.edu.cn; Hao Zheng, hao.zheng@cau.edu.cn;

Yuan Lu, yuanlu@tsinghua.edu.cn

**Supplementary Figures**


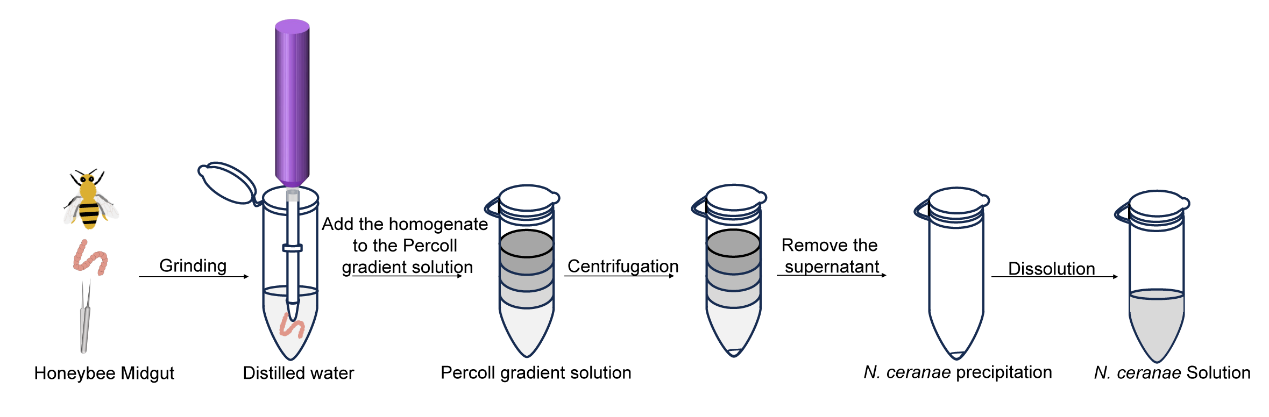


**Fig. S1.** *N. ceranae* isolation. All the gut of the bees were removed with sterilized forceps, and the midgut and colon were cut with scissors. The midgut was placed in 100 μl of distilled water and ground. The midgut homogenate was then added to the Percoll gradient solution and centrifuged at 5000 g for 15 min at 4°C. After centrifugation, the supernatant was removed with a pipette gun, leaving the white precipitate. The white precipitate was added to 100 μl of pure water to obtain the *N. ceranae* solution.


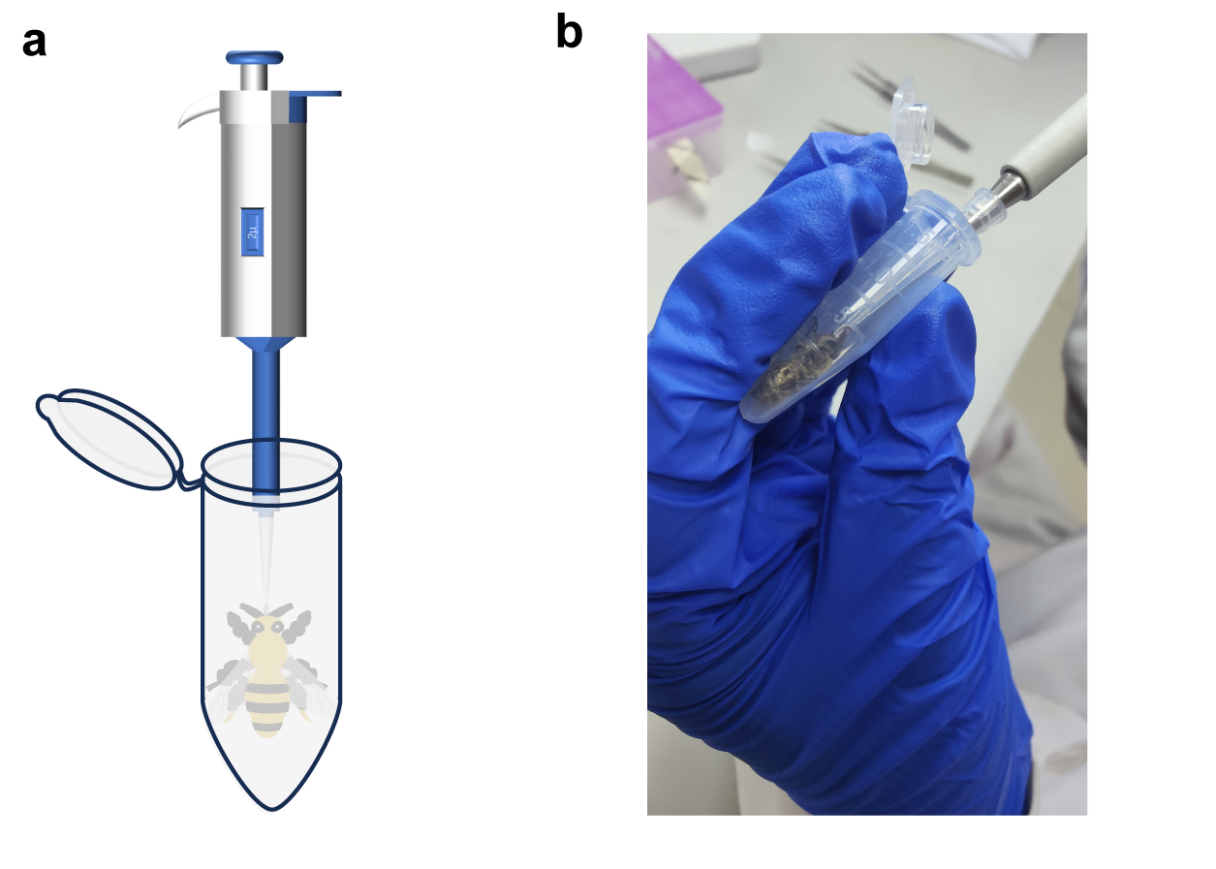


**Fig. S2.** *N. ceranae* infection. Before each infection, bees were starved for 2 h. Then 2 μl of a 50% sucrose solution containing 10^5^ spores was aspirated with a pipette gun and punched out at the mouthparts of the bees, ensuring that the bees ingested the 2 μl of sucrose solution completely.


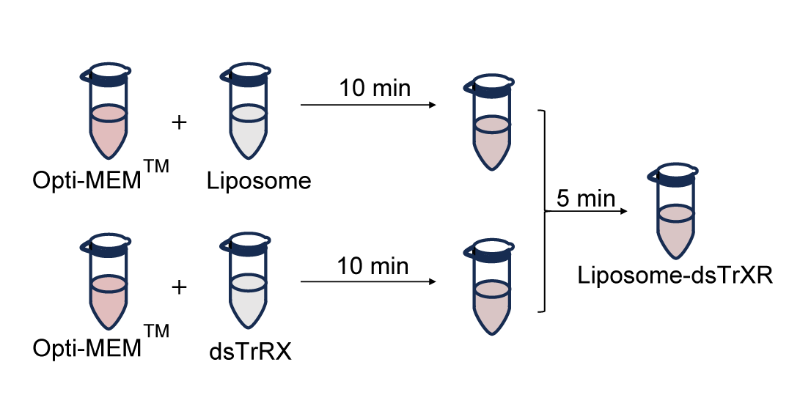


**Fig. S3.** Liposome configuration.


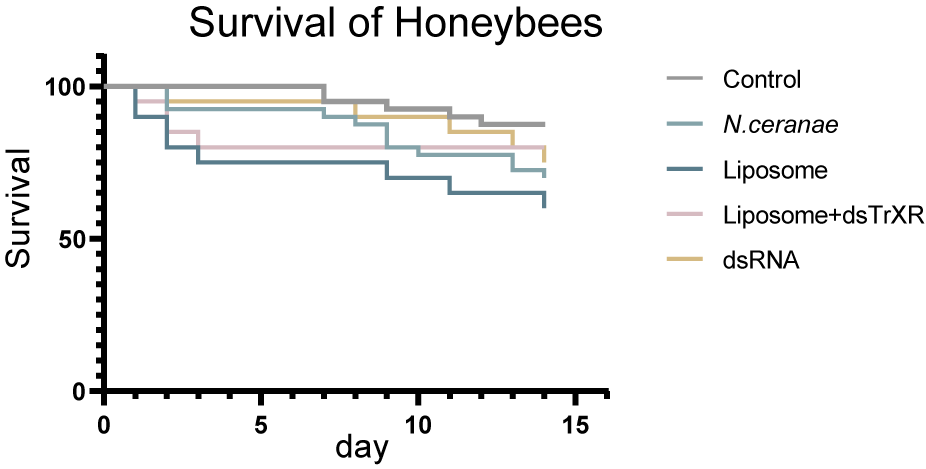


**Fig. S4.** Honeybee survival curve. After infection with *N. ceranae*, the survival of the bees was recorded daily. The results showed that neither liposomes nor *N. ceranae* had a significant effect on the survival of the bees within 14 days.

**Supplementary Table**

**Table S1** Primer sequences

| DNA | Forward | Reverse | Purpose |
| --- | --- | --- | --- |
| Thioredoxin reductase  (*N. ceranae*) | TAATACGACTCACTATAGGGAGGAGGAGACTCTGCTATGGA | TAATACGACTCACTATAGGGCGCAGTCGTACAATCCTCGT | RNAi |
| β-tubulin  (*N. ceranae*) | AGAACCAGGAACGATGGAGA | TCCTTGCAAACAATCTGCAC | qPCR |
| Thioredoxin reductase  (*N. ceranae*) | GTGGTCCTGCAGCCTATTCT | TACTTCCTACCATCCCGCCT | qPCR |
| SSU rRNA  (*N. ceranae*) | AGAAACTACAACAGCATCACTGGGA | AGTGAATATTCCAATTCCCAACGACTT | *Nosema ceranae* |
